# Phytochemical studies for quantitative estimation of iridoid glycosides in *Picrorhiza kurroa* Royle

**DOI:** 10.1186/s40529-016-0121-2

**Published:** 2016-02-26

**Authors:** Phalisteen Sultan, Arif Jan, Qazi Pervaiz

**Affiliations:** CSIR-Plant Biotechnology Division, Indian Institute of Integrative Medicine, Sanatnagar, Srinagar, Kashmir India

**Keywords:** Picroside, Apocyanin, Cucurbitacin, Accessions, Chromatography, Iridoid, Herbal

## Abstract

**Background:**

*Picrorhiza kurroa* Royle commonly known as ‘Kutki or Kutaki’ is an important medicinal plant in Ayurvedic system of medicine and has traditionally been used to treat disorders of the liver and upper respiratory tract. The plant is the principle source of iridoid glycosides, picrosides I, II and kutkoside used in various herbal drug formulations mainly as strong hepatoprotective and immune-modulatory compound. The species has become endangered to near extinction due to the unregulated collection from the wild, slower plant growth and ecological destruction of natural habitats. There is a severe shortage of plant material, while the market demand is ever increasing. Hence, it is very important to apply a simple and precise analytical method to determine and validate the concentration of the major bioactive constituents in different populations of this plant species for development of a high yielding chemotype for large scale production and its commercial exploitation on scientific lines.

**Results:**

This study assessed and validated a fast and reliable chromatography method for the determination of picroside-I and picroside-II in different populations of this priortized medicinal plant species. Separation and resolution of picrosides was carried out on a reversed phase (C-18) column by using a mobile phase of methanol and water (40:60 v/v). The detection of picrosides was carried out at 270 nm. The average levels of these two major marker compounds in all the seven accessions showed significant quantitative variation (ANOVA, p < 0.05) between mean levels of marker compounds and their accumulation in different parts of the plant viz. roots, rhizomes and leaves. The highest content of pk-I was found in the accession from Gurez altitude (3750 masl) while the highest content of pk-II was found in accession from Keller (Shopian) altitude (3300 masl) demonstrate that picroside accusation is directly correlated with altitudinal variation. The method was validated in terms of linearity, accuracy and precision (within- and between-assay variation).

**Conclusion:**

A simple chromatographic method with the ability to separate both the major chemical constituents effectively in different herbal extracts of *P. kurroa* and other related species has been standardized and validated, which is more suitable for regular and normal analysis of picrosides in different herbal formulations. The paper accomplish that picroside concentration in different samples showed significant variation based on altitude and other agroclimatic factors, which can be useful in the selection and collection of superior genotypes with higher concentration of these marker compounds.

## Background

**Fig. 1:**
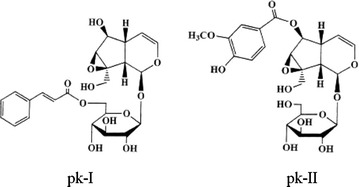
Chemical structure of pk-I and pk-II

Plant based molecules are continuously gaining wide spread acceptance due to their effective therapeutic properties (Dubey et al. 2004). A large number of plant derived molecules of pharmaceutical interest accumlates in plants as secondary metabolites. Realizing the threat of extinction of plants like *P. kurroa* and importance of phytoceuticals produced by such plants, attention has been focused towards cultivation of such endangered plant species involving conventional and biotechnological interventions. *Picrorhiza kurroa* Royle ex. Benth (Scrophulariaceae) is a fast depleting high value medicinal plant, endemic to alpine Himalayan mountains (2700 and 5000 masl) distributed from Kashmir to Sikkim. In view of these facts, it was felt immensely to focus on marker guided phytochemical investigation of this high value medicinal plant, which yield clinically proven hepatoprotective and immune modulating glycosides in its underground parts (Anonymous 1996; Gupta et al. 2006). *Picrorhiza kurroa* an endemic plant of the alpine Himalayan range of India also known as kutki, is mainly found in the north western Himalayan region from Kashmir to Kumaun and Garhwal regions in India and Nepal. *Picrorhiza kurroa* yield a crystalline product called ‘kutkin’ which is a mixture of two major C9 iridoid glycosides such as picroside I and II and kutkoside (Fig. 1) (Kumar et al. 2004). Hepatoprotective, anticholestatic, antiulcerogenic, antiasthematic, antidiabetic, anti-inflamatory and immune regulatory activities have already been ascribed to these glycosides for which *P. kurroa* plant as whole and underground parts in particular finds applications as major component in several Indian herbal préparations (Ram 2001; Thyagarajan et al. 2002). Herbal preparations of *P. kurroa* are used for the treatment of traditional as well as in modern system of medicine as purgative, brain tonic, stomachic, dyspepsia and antiallergic (Hussain 1984). It is also reported to have anticancer activity (Joy et al. 2000) and extracts can be used as selective enhancers of neuron growth (Li et al. 2000, 2002). *Picrorhiza kurroa* extracts can be of high therapeutic value in treating viral hepatitis (Mehrotra et al. 1990).

In the present communication, we have described a rapid and sensitive HPLC method for picroside estimation and effect of altitude on picroside content in some core collections of *P. kurroa* collected from different high altitude ecozones of north western Himalyas viz. Toshimadan (3450 masl), Razdan pass, Gurez (3750 masl), Sonamarg (2799 masl), Gulmarg (3275 masl), Veerinag (3050 masl) and Keller forests, Shopian (3300 masl). We tried in bringing the prospect of achievable, root-derived phytoceuticals in higher concentration and commercial cultivation of *P. kurroa* as a step closer to stop indiscrimnate industrial exploitation of such high value plant resources. We also highlighted and demonstrated that picroside content in tissues vary with the geographical distribution along the North Western Himalayan range. This technique would facilitate large scale clonal production and efforts to improve the secondary metabolism of this prioritized medicinal plant species. This communication highlight detection of two major chemical constituents, pk-I and pk-II for development and validation of an assay method for relative estimation of both these marker compounds in *P. kurroa* collected from seven different geographical ecozones of North Western Himalayas.

## Methods

### Survey, collection and authentication of plant material

*Picrorhiza* plants growing wild in their natural habitat were collected from high altitude regions of North Western Himalayas. The plant material was authenticated at the Centre of Plant Taxonomy, University of Kashmir, Srinagar. A voucher specimen has been deposited in the repository of the Indian Institute of Integrative Medicine, Srinagar against voucher specimen (voucher no. IIIM/PK/Srinagar).

### Chemicals and reagents used

The standard picroside I and II compounds used in the present investigation were isolated in the Natural Product Chemistry division by routine chromatography techniques. Identity and purity was confirmed by chromatographic methods like (TLC, HPLC) and spectral (IR, 1D- and 2D-NMR). Solvents were of HPLC grade and purchased from Ranbaxy Fine Chemicals Limited (Okhla, New Delhi, India). The structures were confirmed by their UV, MS, _1_H NMR and C^13^ NMR data compared with the authentic data from literature. Acetonitrile of HPLC grade (Aldrich, USA) and Millex syringe filter unit were purchased from Reagent, New Delhi, India. Water for preparation of samples and HPLC–DAD analysis was deionized by a Milli-Q purification system with a 0.2 mm fiber filter (Barnstead, CA, USA).

### Moisture analysis

For uniform and standard quantification of biomarkers in plant samples, it is necessary to remove the moisture content in the test sample. To remove the moisture content, the material was dried in hot air oven for 3 h at 50 °C and the process was repeated several times until a uniform dried weight of the sample was obtained. Moisture content was further analyzed in 1.0 g of the dried powdered plant material by the help of Moisture analyzer (Sailorius MA 100).

### Preparation of herbal extracts

The rhizomes and leaf samples were taken from all the seven accessions grown at three different locations viz. gene bank, IIIM, Srinagar, field Station Bonera (Pulwama) and Yarikha (Gulmarg), J&K, India. The air dried plant material was finely powdered and stored at 4 °C. A known quantity of finely powdered sample was weighed into a 250 ml conical flask and subjected to sequential cold extraction using methanol and water in the ratio of 1:1 as extraction solvents while stirring at room temperature. Contents of the flask were squeezed through muslin cloth and the filtrate from aqueous extract was filtered using whatman filter paper. The extraction process was repeated thrice (2–3 h stirring each time). The extracts from each of the sample were evaporated under reduced pressure to give residues in different amounts.The yield of the extract was approximately 10 %. Extract was suspended in HPLC grade methanol in preparatory tubes (5 ml) and used for all experiments.

### Isolation and characterization of marker compounds

The marker compounds picroside I and picroside II were isolated by column chromatography using different solvent systems and percolated over water bath for 3 h at 25 ± 2 °C (Kita et al. 1971; Singh et al. 2005). Use of the solvents in the increasing order of polarity yielded the extracts which helped in clear baseline separation of compounds during LC-UV-DAD-MS analysis. The aqueous extracts were filtered and dried by evaporation at reduced pressure and temperature (40 ± 2 °C). The quality of the residue was analyzed visually by HPTLC. The dried filtrate (200–250 mg) was dissolved in 50 ml methanol to yield picroside I and II using column chromatography. The residue obtained after solvent evaporation was passed through 900-g silica gel (60–120 mesh) column. The column chromatography of methanolic extract residue in chloroform with increasing polarity of methanol yielded two major marker compounds picroside I [6-0-trans-cinnamoyl catalpol] and picroside II [6-O-vanilloyl catalpol]. The isolated compounds were characterized by recording melting point using DSC, UV, IR and Mass spectra and identified by comparing with the reported data (Singh et al. 1993).

### Preparation of stock solutions

Stock solutions of both the reference marker compounds, picroside I and picroside II (1000 mg/ml) were prepared in HPLC grade water. The concentration (μg ml^−1^) of each compound was 69 for pk-I and 125 for pk-II. The mixed solution was filtered through a Millipore filter (0.2 mm) before injection onto the HPLC system. The solution was prepared by accurately adding appropriate volume of each of the prepared standard solution. 10 µl of this mixed solution was injected in the column which exhibited different retention times for both the marker compounds. All the solutions were stored in refrigerator at 4 °C and kept at room temperature before use. On the same day of analysis, the precision as well as reproducibility of the method was observed. The peaks of the sample solution were identified by comparison of the retention time with those corresponding authentic samples run individually.

### Calibration solutions

In order to establish the linear detection range for each compound, individual standard solutions were prepared in mobile phase in 250 ml measuring flask. Aliquots of these solutions were diluted and analyzed to determine method linearity. Limit of quantification (LOQ) values were estimated from serial dilution and analyzed for each sample. Triplicate 10 μl injections were made for each compound to see the reproducibility of the detector response at each concentration level. The peak area of each compound was plotted by running different concentrations to obtain the calibration graph. The five concentrations of each compound were subjected to regression analysis to calculate calibration equation and correlation coefficients.

### Analytical HPLC conditions

**Fig. 2:**
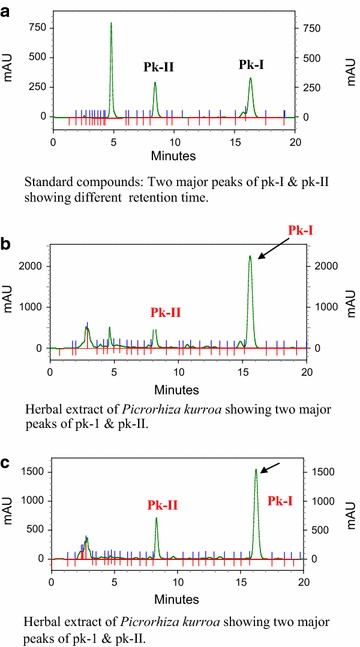
Chromatograms of **a** standards. **b** and **c** resolution of picroside-I and picroside-II in two different herbal preparations

The HPLC analysis was performed on a ThermoFinnigan HPLC machine equipped with auto sampler, column apartment and UV detector for the visual confirmation of the presence of marker compounds in the extract. Acquiring and analysis of data was controlled by Shemstation software (Agilent Tech, USA). A RP-18 column (3 mm × 150 mm) from E. Merck was used at 30 °C temperature. Separation was done using methanol and water (40:60) at a flow rate of 0.7 ml/min with injection volume of 4 ul for each sample and UV detection was set at 270 nm. Prior to use, solvents were filtered through a 0.22 mm diameter membrane filters. Equal volume of the standard solution were mixed and injected in the HPLC system in volumes of 2, 4, 6, 8 and 10 µl for plotting calibration curves. Solutions were injected in triplicate and the calibration curve was plotted by plotting concentration value of each anlyte. Satisfactory separation was obtained as shown in the chromatograms (Fig. 2). Quantification and linearity of the HPLC method was carried out for picroside content in different samples of *P. kurroa*. The percentage of the picrosides was calculated by calibration curve using peak height and peak area ratio. ICH guidelines were followed for the validation of the analytical methods for precision, repeatability and accuracy.

### Linearity and accuracy

Linearity was determined by analyzing the standard solutions of both the picrosides pk-I and pk-II at five levels. The least square regression equation and correlation coefficient were used for assessing the linearity. Recovery and accuracy was assessed by the addition of known amounts of picroside I and picroside II to the pre-analyzed sample at three different concentration levels. The applicability of the extraction procedure was confirmed by recovery experiments of 99.9 and 100 % and the relative standard deviations (RSD) calculated for all samples proved to be less than 2 %.

## Results and discussion

The major iridoid glycosides of all the seven analyzed populations of *P. kurroa* collected from different eco-geographicai locations viz. Toshimadan (3450 masl), Razdan pass, Gurez (3750 masl), Sonamarg (2799 masl), Gulmarg (3275 masl), Veerinag (3050 masl) and Keller–Shopian (3300 masl) of Kashmir Himalayas in India observed the accumulation of picroside I and picroside II in varying amounts (Table 2). The use of cold solvent extraction provided speedy, efficient and reproducible results. The use of cold extraction was essential to selectively isolate and enrich the picrosides from the total extract. The results signify that there were not only remarkable variation in the total amounts of picroside I and picroside II but also revealed their relative contents in different habitats. Out of the seven analyzed accessions, the highest content of pk-I (5.18 %) was found in the accession from Gurez valley and lowest content (2.78 %) in Gulmarg population, while the specimen from Keller (Shopian) contained highest content of pk-II (5.39 %) and lowest (2.53 %) was found in specimen from Sonamarg ecozone. Regression analysis of the calibration curves of the two markers gave linear equations with a regression coefficient of 0.999. Recoveries of markers were not less than 99 % (Table 1). Analysis of all the 180 samples showed detectable concentration of both pk-I and pk-II (Fig. 2) in almost all samples at (mg/g) levels. At such low level of detection, the identity of each peak had to be confirmed by matching its PDA generated UV spectrum with that of its respective reference standard.

**Table 1:** Recovery of picroside-I and picroside-II

Standard	Spiked (mg)	Found (mg)	Recovery %	Recovery % (R.S.D)
Picroside-I	0.0038	0.0080	103.6	*101.3 (2.54)*
	0.0046	0.0086	104.3	
	0.1771	0.3944	96.1	
Picroside-II	0.0680	0.1333	100.2	*98.63 (1.45)*
	0.807	0.1423	99.2	
	0.0462	0.1027	96.5	

The mean levels of the two marker compounds in representative samples from all the seven collected populations showed significant quantitative variation (ANOVA, p < 0.05) between mean levels of both the marker compounds (Table 2). There was also significant difference in the active content of samples collected from different ecozones, which suggests that environmental and genetic factors have played a critical role in determining the chemical profile of such high altitude plant species. The observed differences were significant for most of the comparisons, as determined by ANOVA analysis (p < 0.05). The quantitative variation observed in the accumulation of these bioactive markers focuses on the importance of standardization and quality evaluation of medicinal formulations based on *P. kurroa*. Picroside levels vary considerably along with varied amounts in the naturally growing *Picrorhiza kurroa* populations collected from different geographical locations. The content of picroside found in the plant species was affected by various factors such as growth conditions, seasonal and geographical variations as well as breeding behaviour. HPLC chromatograms of the herbal extracts showed many resolved peaks. The peaks were identified by comparison of their retention time to that of standard picrosides run simultaneously for linear calibration curve. Linear regression was used to establish the calibration curve.

**Table 2:** Chemical profiling of various herbal extracts of *Picrorhiza kurroa* based on two major marker compounds

Location	Date of analysis	Code	Percentage of marker compounds											
			Picroside I (% Dry wt. basis)						Picroside-II (% Dry wt. basis)					
			R1	R2	R3	R4	R5	Mean ± SD	R1	R2	R3	R4	R5	Mean ± SD
Toshimadan	Dec–Jan, 2004	Pk-Ts	2.17	2.37	2.18	1.98	2.20	*2.18* ± *0.13*	1.19	2.33	2.30	1.30	2.23	*1.67* ± *0.57*
Toshimadan	June–July, 2004	Pk-Ts	3.84	3.09	4.06	2.95	2.89	*3.36* ± *0.54*	4.46	3.33	3.34	3.03	4.21	*3.47* ± *0.62*
Toshimadan	Dec–Jan, 2005	Pk-Ts	2.36	3.25	3.71	3.15	4.58	*3.41* ± *0.81*	4.12	4.35	5.03	4.26	4.29	*4.41* ± *0.35*
Toshimadan	June–July, 2005	Pk-Ts	2.35	2.00	2.96	2.04	1.95	*2.26* ± *0.4*	3.21	4.51	3.43	4.11	3.59	*3.57* ± *0.53*
Toshimadan	Dec–Jan, 2006	Pk-Ts	3.76	4.59	3.61	5.33	4.36	*4.53* ± *0.69*	5.91	6.32	5.24	5.79	6.14	*5.88* ± *0.41*
Toshimadan	June–July, 2006	Pk-Ts	3.66	3.96	2.22	3.41	3.35	*3.32* ± *0.66*	4.72	3.68	5.02	3.84	5.09	*5.65* ± *0.66*
								*3.17* ± *0.86*						*4.10* ± *1.56*
Gurez	Dec–Jan, 2004	Pk-Gr	5.06	5.99	4.89	5.23	3.68	*5.12* ± *0.83*	4.5	3.10	3.02	4.1	3.23	*3.81* ± *0.66*
Gurez	June–July, 2004	Pk-Gr	2.06	2.11	2.47	4.03	3.98	*2.93* ± *0.99*	1.09	2.02	1.36	1.84	2.44	*1.75* ± *0.53*
Gurez	Dec–Jan, 2005	Pk-Gr	6.21	6.19	6.72	8.76	6.77	*6.93* ± *1.05*	4.64	5.19	6.10	6.86	5.81	*5.72* ± *0.85*
Gurez	June–July, 2005	Pk-Gr	2.89	2.21	4.32	4.97	4.61	*3.80* ± *1.18*	2.87	2.47	3.25	3.51	4.20	*3.60* ± *0.65*
Gurez	Dec–Jan, 2006	Pk-Gr	3.40	4.78	3.39	4.99	5.84	*4.48* ± *1.06*	4.81	6.70	5.99	5.92	5.43	*5.77* ± *0.70*
Gurez	June-July, 2006	Pk-Gr	6.61	7.47	8.29	7.85	8.98	*7.84* ± *0.88*	6.25	7.61	6.68	5.98	6.23	*6.55* ± *0.64*
								*5.18* ± *1.87*						*4.53* ± *1.79*
Sonamarg-B	Dec–Jan, 2004	PkS-B	5.96	5.83	4.86	4.82	6.78	*5.65* ± *0.82*	1.25	1.41	0.87	1.03	1.19	*1.15* ± *0.20*
Sonamarg-B	June–July, 2004	PkS-B	2.79	2.31	4.02	2.02	3.16	*2.86* ± *0.78*	4.06	4.95	5.07	4.41	6.47	*4.99* ± *0.92*
Sonamarg-B	Dec–Jan, 2005	PkS-B	4.03	5.91	5.99	7.38	6.79	*6.02* ± *1.26*	1.18	1.12	0.91	1.15	1.74	*1.22* ± *0.30*
Sonamarg-B	June–July, 2005	PkS-B	1.31	1.42	1.23	2.20	1.19	*1.47* ± *0.41*	3.91	5.18	5.86	5.74	6.76	*5.49* ± *1.04*
Sonamarg-B	Dec–Jan, 2006	PkS-B	1.01	0.84	1.35	0.76	0.84	*0.96* ± *0.23*	1.37	1.24	1.09	1.56	0.74	*1.20* ± *0.30*
Sonamarg-B	June–July, 2006	PkS-B	0.79	1.13	0.78	0.49	1.01	*0.82* ± *0.24*	0.87	1.96	0.78	1.31	0.83	*1.15* ± *0.49*
								*2.95* ± *2.34*						*2.53* ± *2.10*
Gulmarg	Dec–Jan, 2004	Pk-G	1.20	1.68	0.93	1.88	3.07	*1.75* ± *0.82*	3.71	4.36	2.12	2.18	2.13	*2.90* ± *1.06*
Gulmarg	June–July, 2004	Pk-G	2.19	3.11	2.17	2.51	3.27	*2.65* ± *0.51*	3.91	2.33	4.04	3.01	2.86	*3.23* ± *0.72*
Gulmarg	Dec–Jan, 2005	Pk-G	2.52	3.72	2.55	3.42	2.59	*2.96* *±* *0.56*	2.53	5.44	5.45	4.34	4.84	*4.78* ± *1.20*
Gulmarg	June–July, 2005	Pk-G	2.09	2.24	2.56	1.45	3.31	*2.33* *±* *0.68*	2.61	1.84	2.61	1.23	1.91	*2.09* ± *0.58*
Gulmarg	Dec–Jan, 2006	Pk-G	2.79	3.09	3.11	4.21	3.85	*3.41* ± *0.59*	4.14	4.05	3.48	3.60	4.23	*3.90* ± *0.33*
Gulmarg	June–July, 2006	Pk-G	3.12	4.09	3.78	2.68	4.37	*3.58* ± *0.69*	4.92	4.93	3.22	4.27	5.21	*4.51* ± *0.79*
								*2.78* ± *0.68*						*3.56* ± *1.02*
Sonamarg-Y	Dec–Jan, 2004	PkS-Y	1.20	2.06	1.23	3.24	2.37	*2.02* ± *0.85*	2.1	1.49	2.47	2.91	2.78	*2.35* ± *0.57*
Sonamarg-Y	June–July, 2004	PkS-Y	4.98	2.95	3.84	5.95	4.43	*4.43* ± *1.13*	2.82	3.94	2.37	4.94	3.78	*3.57* ± *1.00*
Sonamarg-Y	Dec–Jan, 2005	PkS-Y	7.0	6.41	5.92	5.91	6.81	*6.41* ± *0.49*	3.93	2.46	2.81	3.47	4.68	*3.47* ± *0.88*
Sonamarg-Y	June–July, 2005	PkS-Y	3.55	5.02	3.28	5.84	4.41	*4.42* ± *1.05*	4.58	3.91	2.03	4.59	4.69	*3.96* ± *1.12*
Sonamarg-Y	Dec–Jan, 2006	PkS-Y	4.40	5.93	5.11	4.77	6.19	*5.28* ± *0.76*	4.61	6.5	6.98	4.88	6.86	*5.88* ± *1.13*
Sonamarg-Y	June–July, 2006	PkS-Y	7.55	7.41	4.92	7.65	6.88	*6.95* ± *1.13*	5.81	5.94	7.16	8.23	7.96	*7.02* ± *1.11*
								*4.91* ± *1.75*						*4.37* ± *1.73*
Veerinag	Dec–Jan, 2004	PkV	3.06	4.92	4.09	5.07	5.01	*4.43* ± *0.86*	3.01	3.65	3.68	4.41	4.0	*3.75* ± *0.51*
Veerinag	June–July, 2004	PkV	5.49	6.85	4.68	5.11	6.12	*5.65* ± *0.85*	3.94	4.68	5.33	6.42	5.23	*5.12* ± *0.91*
Veerinag	Dec–Jan, 2005	PkV	1.02	1.81	2.19	2.17	2.66	*1.97* ± *0.61*	2.41	2.34	2.79	1.92	2.9	*2.47* ± *0.39*
Veerinag	June–July, 2005	PkV	3.91	5.53	3.71	3.77	4.28	*4.24* ± *0.75*	2.44	3.32	2.22	2.85	3.12	*2.99* ± *0.45*
Veerinag	Dec–Jan, 2006	PkV	2.41	3.81	4.12	3.26	4.90	*3.70* ± *0.93*	2.40	2.18	2.89	3.86	2.92	*2.85* ± *0.64*
Veerinag	June–July, 2006	PkV	3.60	3.82	4.94	4.29	3.85	*4.10* ± *0.53*	7.12	8.04	6.39	7.61	6.04	*7.04* ± *0.82*
								*4.01* ± *1.19*						*4.03* ± *1.74*
Keller	Dec–Jan, 2004	PkK	2.11	1.91	2.12	2.47	3.09	*2.34* ± *0.46*	6.50	8.90	6.21	5.78	6.96	*6.87* ± *1.21*
Keller	June–July, 2004	PkK	2.12	1.33	2.11	2.09	1.25	*1.78* ± *0.44*	2.0	2.51	2.41	2.15	1.68	*2.15* ± *0.33*
Keller	Dec–Jan, 2005	PkK	2.24	2.92	2.99	3.56	3.29	*2.98* ± *0.49*	4.89	4.09	3.23	5.41	4.33	*4.41* ± *0.82*
Keller	June–July, 2005	PkK	2.31	3.81	2.12	3.05	4.86	*3.23* ± *1.12*	5.03	4.7	4.28	5.52	5.22	*4.75* ± *0.47*
Keller	Dec–Jan, 2006	PkK	3.19	3.41	3.09	5.17	4.94	*3.96* ± *1.00*	7.87	8.21	8.06	5.09	8.97	*7.04* ± *1.48*
Keller	June–July, 2006	PkK	3.03	2.28	3.41	1.87	3.81	*2.88* ± *0.79*	8.3	6.5	6.6	6.39	7.86	*7.13* ± *0.88*
								*2.86* ± *0.74*						*5.39* ± *1.99*

To assess the precision of this chromatography method, the results of the recoveries of pk-I and pk-II ranged from 98.6 to 101.3 %. The extraction efficiency, three times work up was sufficient since it allowed over 95 % extraction of both the marker compounds. The content of the two marker constituents in rhizomes and leaves of *P. kurroa* growing in different locations was also analysed, demonstrating that all of these phytochemicals in the target plant are interestingly dependent of the locality. The proposed. HPLC as well as extraction method is simple, reliable, robust and sensitive and can detect chemical constituents in very small quantities.

## Conclusion

A simple, sensitive and rapid extraction and HPLC method has been developed and validated for detection and determination of various chemical constituents in very small quantities. The method is simple, precise and accurate with the ability to separate all the chemical constituents effectively, which is more suitable for regular and normal analysis of picrosides in different drug formulations and screening of plant specimens for genotype assessment.
